# Lower pre-conditioning absolute lymphocyte counts are associated with worse outcomes in haploidentical stem cell transplantation with myeloablative regimen in children

**DOI:** 10.3389/fimmu.2025.1552263

**Published:** 2025-03-25

**Authors:** Kai Cui, Senlin Zhang, Yueke Du, Yutan Chai, Mingchu Liang, Shaoyan Hu, Jie Li

**Affiliations:** ^1^ Department of Hematology and Oncology, Children’s Hospital of Soochow University, Suzhou, China; ^2^ Department of Nephrology and Immunology, Children’s Hospital of Soochow University, Suzhou, China; ^3^ Jiangsu Pediatric Hematology and Oncology Center, Suzhou, China

**Keywords:** hematopoietic stem cell transplantation, haploidentical, anti-thymocyte globulin, absolute lymphocyte counts, pediatric

## Abstract

**Background:**

Anti-thymocyte globulin (ATG) is frequently administered for preventing graft-versus-host disease (GVHD) in allogeneic hematopoietic stem cell transplantation (allo-HSCT). In patients with low absolute lymphocyte count (ALC) before conditioning, weight-based dosing of ATG may cause overexposure, negatively impacting prognosis.

**Method:**

Clinical data of patients with hematological malignancies undergoing haploidentical HSCT (haplo-HSCT) at the Children’s Hospital of Soochow University from January 2020 to June 2023 were collected. This study primarily aims to investigate the association between pre-conditioning ALC and clinical outcomes in pediatric acute leukemia or myelodysplastic syndromes patients receiving myeloablative haplo-HSCT.

**Results:**

We included 130 patients treated at the Children’s Hospital of Soochow University from January 2020 to June 2023. According to the cutoff of 500/μl, patients were divided into high and low ALC groups. Patients in the high ALC group experienced a higher incidence of II-IV acute GVHD (30.2% versus 13.6%, *P* = 0.034), 3-year overall survival (OS) and relapse-free survival (RFS) rates (OS: 88.5% ± 3.7% versus 66.9% ± 7.9%, *P* = 0.013; RFS: 81.4% ± 4.1% versus 56.5% ± 8.1%, *P* < 0.001), and lower cumulative incidence of relapse (11.3% versus 27.4%, *P* = 0.013). Pre-conditioning ALC < 500/μl independently predicted worse OS, RFS, and higher relapse risk in multivariate analysis. However, there was no significant difference in immune reconstitution between the two groups.

**Conclusion:**

Pre-conditioning ALC was a significant prognostic factor in pediatric patients undergoing myeloablative haplo-HSCT. Further research is needed to explore whether pre-conditioning ALC can serve as a reference for adjusting ATG dosing.

## Introduction

Allogeneic hematopoietic stem cell transplantation (allo-HSCT) serves as a curative approach for acute leukemia and myelodysplastic syndrome (MDS). Transplant-related complications, such as graft-versus-host disease (GVHD), and relapse remain major obstacles to long-term survival (1, 2).

To avoid GVHD, anti-thymocyte globulin (ATG), a polyclonal IgG made from horse or rabbit serum, is frequently used to deplete T cells *in vivo* (3). Several studies have proved that ATG can reduce the incidence of acute (aGVHD) and chronic GVHD (cGVHD) and has no effects on the relapse and overall survival (OS) (4, 5).

The most optimal dosing strategy for ATG remains controversial. Currently, the common dosing strategy is based on recipient body weight in pediatric patients. However, the clearance rate differs among pediatric patients, leading to highly variable ATG exposure (6, 7). Insufficient dosing may be ineffective in reducing GVHD incidence, whereas excessive dosing is linked to worse clinical outcomes (8). Recently, studies have found that ATG clearance is related to absolute lymphocyte count (ALC). Thus, patients with lower ALC levels at the time of transplantation may have higher residual circulating serum ATG levels, potentially raising the incidence of mortality (9, 10).

However, most studies focus on human leukocyte antigen matching transplants and yielded inconsistent results. Our analysis focused on investigating the role of pretreatment ALC in predicting post-transplant outcomes after myeloablative haploidentical HSCT (haplo-HSCT) with ATG.

## Method

### Patients

Patients with hematological malignancies treated with myeloablative haplo-HSCT from January 2020 to June 2023 were included. Peripheral blood stem cells or combined bone marrow stem cells were used as graft sources. The inclusion criteria were as follows: (a) diagnosed with acute myeloid leukemia (AML), acute lymphoblastic leukemia (ALL), or myelodysplastic syndrome (MDS); (b) no prior HSCT; (c) no prior CAR-T cell therapy or lymphocyte infusion; (d) able to acquire pre-conditioning ALC.

### Procedures

All patients received cyclophosphamide (60 mg/kg, days -3 to -2) and busulfan (3.2 mg/kg, days -7 to -4) or total body irradiation (TBI, 4 Gy/day, days -7 to -5) as part of the conditioning regimen. Me-CCNU (250 mg/m²) was given on day -13, along with cladribine (5 mg/m²/day) or fludarabine (30 mg/m²/day) and Ara-C (2 g/m²/day) from days -12 to -8. Rabbit ATG (2.5 mg/kg, 4 days) was administered from days -5 to -2. Starting day +6, granulocyte colony-stimulating factor (5 μg/kg/day) was given until the absolute neutrophil count (ANC) exceeded 1 × 10^9^/L.

GVHD prevention involves a combination of methotrexate (15 mg/m² on days +1 and 10 mg/m² on days +3, +6, and +11) and mycophenolate mofetil (20 to 30 mg/kg/day from days −1 to + 30 and then half the dose for 15 consecutive days) supplemented with cyclosporine and tacrolimus (blood levels: 200–250 ng/mL and 10–15 ng/mL, respectively).

Starting from the conditioning regimen, all patients received prophylactic ganciclovir before stem cell infusion at a dose of 5 mg/kg twice daily, and after infusion, they were given acyclovir (10mg/kg) twice daily. Regular cytomegalovirus (CMV) and Epstein-Barr virus (EBV) DNA testing was performed after transplantation, and ganciclovir (5 mg/kg twice daily) was administered upon viral reactivation until seronegative status was achieved.

### Pre-conditioning ALC

The pre-conditioning ALC was obtained through lymphocyte subsets on day -14. On the morning of day -14 before transplantation, 100 µL of peripheral blood was collected from patients for lymphocyte subset analysis. Data were acquired using a Gallios flow cytometer (Beckman, Los Angeles, CA, USA) and analyzed with FlowJo software to obtain quantitative information on lymphocyte subsets. All samples were collected within the same time window (between 8:00 and 10:00 AM) to minimize diurnal variation.

### Endpoints

Our study primarily aims to investigate the relationship between pre- conditioning ALC and clinical outcomes, including OS, relapses, relapse-free survival (RFS), and non-relapse mortality (NRM). The secondary objective is to investigate GVHD and viral infections. OS was the interval from transplantation to death from any cause. RFS refers to the period from transplantation to relapse or all-caused death. NRM is the death caused by any reason other than relapse. Relapse occurs when leukemia cells comprise over 5% of the bone marrow or when extramedullary leukemia is detected. GVHD was diagnosed and graded based on established standards (11). The diagnostic criteria for CMV-DNA and EBV-DNA seropositivity are a peripheral blood DNA copy number of ≥500 copies/mL in two successive tests.

Neutrophil engraftment is considered when the ANC is ≥0.5 × 10^9^/L for 3 successive days. Platelet engraftment is confirmed when the platelet count remains ≥ 20 × 10^9^/L for 7 successive days without transfusion.

### Statistical analysis

The t-test and Mann-Whitney U test were conducted to assess continuous variables. To assess categorical variables, the Chi-square test or Fisher’s exact test was applied. To depict OS and RFS, Kaplan-Meier curves were employed, with comparisons made by the log-rank test. For outcomes involving competing risks, such as NRM, relapses, GVHD, and infection, Gray’s test was applied to analyze the discrepancies. Pearson’s correlation test was applied to examine the relationship between continuous variables. In addition, using time-dependent Cox regression, univariate analysis was carried out, and variables with a P-value ≤ 0.10 were incorporated into the multivariate analysis. It was regarded as significant if P-value <0.05. R 4.3.3 software was employed for statistical analyses in this study.

## Results

### Patients

The study included 130 patients, 52 males and 78 females, with a median age of 92.5 (2.0-206.0) months. The underlying disease included 88 AML (67.7%), 41 ALL (31.5%) and 1 MDS (0.8%). Most patients (90.8%) reached complete remission (CR) before HSCT. 126 patients had details about minimal residual disease (MRD). Among them, 101 patients tested negative for MRD, and 25 patients tested positive. Regarding the graft source, 47 patients (36.2%) were from peripheral blood stem cells, while 83 patients (63.8%) received a combination of peripheral blood and bone marrow stem cells.

A cutoff of 500/μl was used to classify patients into low and high ALC groups (10). The low ALC group consisted of 44 patients, while the high ALC group included 86 patients. The low ALC group was significantly older than the high ALC group (110.5 ± 50.4 months vs. 90.3 ± 56.1 months, P = 0.028). Aside from this, the two groups showed no statistical differences in sex, underlying disease, MRD, number of chemotherapy, or the infusion dose of CD34+ cells. Patient baseline characteristics can be found in **Table 1**.

**Table 1:** Patient characteristics with high and low pre- conditioning absolute lymphocyte count.

	ALC <500/μL	ALC ≥500/μL	P-value
Age (months)	112.5 (2, 188)	84 (6, 206)	0.028
Sex, n (%)			0.198
Male	21 (47.7)	31 (36.0)	
Female	23 (52.3)	55 (64.0)	
Disease, n (%)			0.420
ALL	12 (27.3)	29 (33.7)	
AML	32 (72.7)	56 (65.1)	
MDS	0	1 (1.2)	
Status, n (%)			0.548
CR	39 (88.6)	79 (91.9)	
Non-CR	5 (11.4)	7 (8.1)	
MRD			0.312
Positive	11 (25.0)	14 (16.3)	
Negative	31 (70.5)	70 (81.4)	
NA	2 (4.5)	2 (2.3)	
TBI			0.395
Positive	5 (11.4)	6 (7.0)	
Negative	39 (88.6)	80 (93.0)	
ABO matched			0.895
matched	23 (52.3)	46 (53.5)	
mismatched	21 (47.7)	40 (46.5)	
Donor-recipient sex match, n (%)			0.288
female to male	5 (11.4)	16 (18.6)	
others	39 (88.6)	70 (81.4)	
Graft source			0.630
PB	14 (31.8)	33 (38.4)	
PB+BM	30 (68.2)	53 (61.6)	
MNC (× 10^8^ /kg)	6.7 (2.1, 14.5)	6.8 (0.7, 21.2)	0.637
CD34+cell (× 10^6^ /kg)	7.3 (2.2, 16.3)	6.9 (0.9, 15.0)	0.663
Engraftment time (days)			
Granulocyte	12.0 (10, 21)	11.5 (9, 20)	0.876
Platelet	11.0 (5, 40)	11.0 (7, 35)	0.805
D-5 ALC	20.0 (0.0, 300.0)	40.0 (0.0, 930.0)	0.046

ALL, acute lymphoblastic leukemia; AML, acute myeloid leukemia; MDS, myelodysplastic syndrome; CR, complete remission; MRD, minimal residual disease; TBI, total body irradiation; PB, peripheral blood; BM, bone marrow; MNC, mononuclear cell; ALC, Absolute Lymphocyte Counts.

### Outcomes

**Figure 1:**
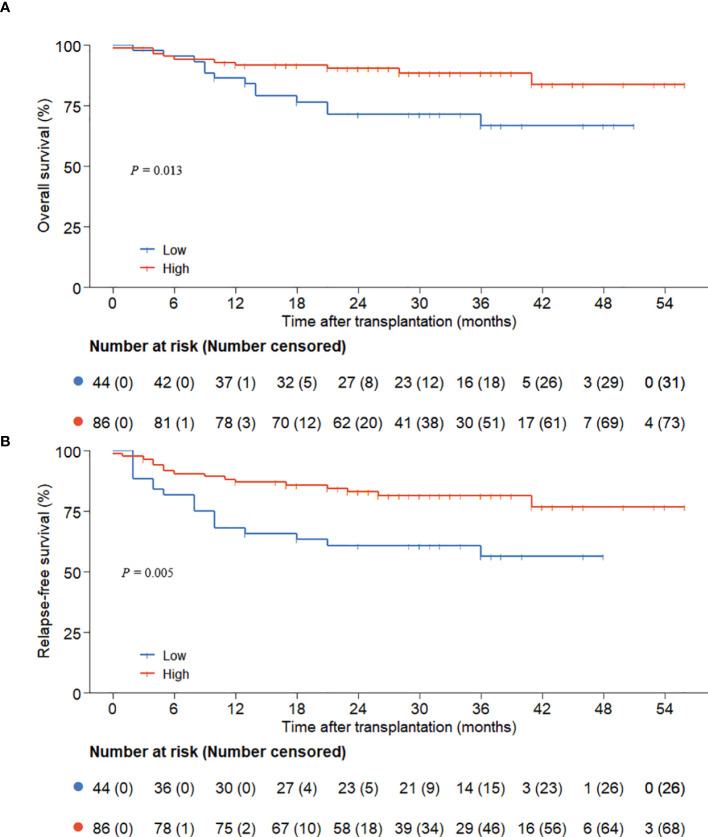
OS and RFS according to preconditioning absolute lymphocyte counts. **(A)** overall survival; **(B)** relapse-free survival.

The follow-up concluded on December 1, 2024, with a median duration of 29 (0.1, 56.0) months. The low ALC group experienced 13 deaths, while the high ALC group had 10 deaths. The 3-year OS and RFS rates were considerably superior in the high ALC group (OS: 88.5% ± 3.7% versus 66.9% ± 7.9%, *P* = 0.013; RFS: 81.4% ± 4.1% versus 56.5% ± 8.1%, P=0.005, **Figures 1A, B**).

Time-dependent Cox regression analysis was conducted to determine if pre-conditioning ALC was an independent risk factor for OS and RFS. In the univariate analysis, the graft dosage of CD34+ cells and ALC < 500/μl were linked to OS (*P* = 0.036 and *P* = 0.017, respectively), while MRD, graft dosage of CD34+ cells, and ALC < 500/μl were linked to RFS (*P* = 0.045, *P* = 0.029, and *P* = 0.006, respectively). However, ALC lacked prognostic significance when analyzed as a continuous variable. Subsequently, multivariate analysis was conducted on variables with a P-value ≤ 0.10. The results indicated that the lower graft dosage of CD34+ cells (OS: *P* = 0.029; RFS: *P* = 0.026) and ALC < 500/μl (OS: *P* = 0.012; RFS: *P* = 0.007) emerged as critical factors influencing both OS and RFS (**Tables 2**, **3**).

**Table 2:** Univariate and multivariate analysis of prognostic factors of OS.

	Univariate Analysis			Multivariate Analysis		
Variable	HR	95% CI	P-value	HR	95% CI	P-value
Sex (male VS female)	0.686	0.302-1.555	0.367			
Age	1.002	0.994-1.010	0.598			
Disease (AML & MDS VS ALL)	0.904	0.394-2.077	0.812			
Disease status (CR VS non-CR)	0.870	0.204-3.711	0.850			
MRD (positive VS negative)	1.597	0.829-3.073	0.161			
TBI (positive VS negative)	1.273	0.298-5.436	0.744			
Blood-type match (mismatched vs matched)	0.742	0.321-1.716	0.486			
Donor-recipient sex match (female to male vs others)	0.889	0.301-2.628	0.831			
Graft source (PB+BM vs PB)	0.722	0.311-1.679	0.450			
Graft Dose						
MNC	1.014	0.864-1.191	0.862			
CD34	0.836	0.707-0.989	**0.036**	0.824	0.692-0.981	**0.029**
ALC (high VS low)	0.365	0.160-0.834	**0.017**	0.348	0.152-0.797	**0.012**
ALC (continuous)	0.999	0.998-1.000	0.158			

OS, overall survival; HR, hazard ratio; CI, confidence internal; ALL, acute lymphoblastic leukemia; AML, acute myeloid leukemia; MDS, myelodysplastic syndrome; CR, complete remission; MRD, minimal residual disease; TBI, total body irradiation; PB, peripheral blood; BM, bone marrow; MNC, mononuclear cell; ALC, Absolute Lymphocyte Counts. Bold values: P ≤ 0.10 in univariate analysis and P < 0.05 in multivariate analysis.

**Table 3:** Univariate and multivariate analysis of prognostic factors of RFS.

	Univariate Analysis			Multivariate Analysis		
Variable	HR	95% CI	P-value	HR	95% CI	P-value
Sex (male VS female)	0.683	0.348-1.340	0.268			
Age	0.999	0.993-1.006	0.871			
Disease (AML & MDS VS ALL)	0.950	0.475-1.900	0.885			
Disease status (persistent VS remission)	2.102	0.870-5.080	**0.099**	1.155	0.423-3.155	0.778
MRD (positive VS negative)	1.703	1.012-2.868	**0.045**	1.610	0.860-3.014	0.137
TBI (positive VS negative)	1.267	0.387-4.149	0.695			
Blood-type match (mismatched vs matched)	0.923	0.469-1.816	0.816			
Donor-recipient sex match (female to male vs others)	0.729	0.281-1.889	0.515			
Graft source (PB+BM vs PB)	1.099	0.534-2.259	0.798			
Graft Dose						
MNC	0.977	0.852-1.120	0.740			
CD34	0.861	0.753-0.985	**0.029**	0.846	0.730-0.980	**0.026**
ALC (high VS low)	0.386	0.196-0.760	**0.006**	0.391	0.197-0.778	**0.007**
ALC (continuous)	1.000	0.999-1.000	0.291			

RFS, relapse-free survival; HR, hazard ratio; CI, confidence internal; ALL, acute lymphoblastic leukemia; AML, acute myeloid leukemia; MDS, myelodysplastic syndrome; CR, complete remission; MRD, minimal residual disease; TBI, total body irradiation; PB, peripheral blood; BM, bone marrow; MNC, mononuclear cell; ALC, Absolute Lymphocyte Counts. Bold values: P ≤ 0.10 in univariate analysis and P < 0.05 in multivariate analysis.

**Figure 2:**
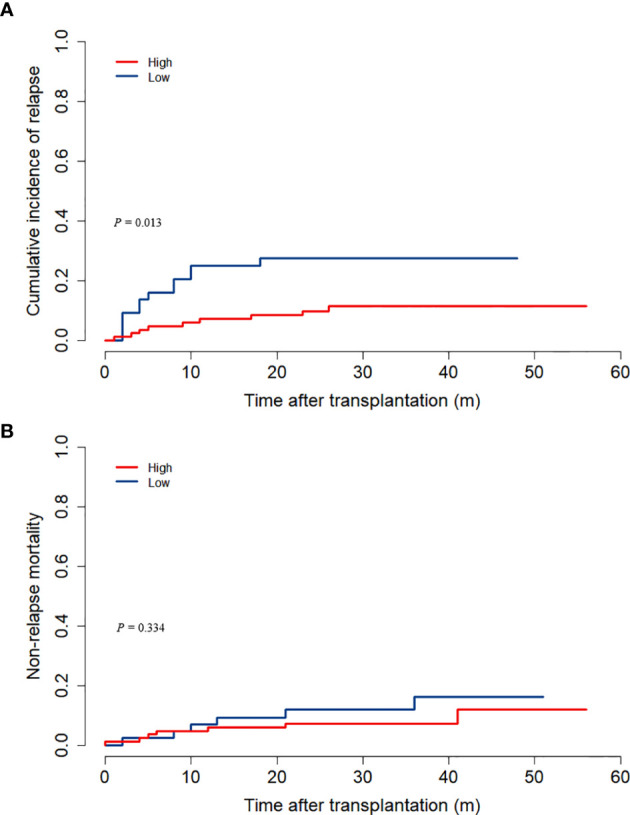
The cumulative incidences of relapse and NRM according to preconditioning absolute lymphocyte counts. **(A)** relapse; **(B)** NRM.

12 patients in the low ALC group experienced relapses, and 9 patients in the high ALC group relapsed. As death was regarded as a competing event, the cumulative incidence of relapse was elevated in the low ALC group (27.4% versus 11.3%, *P* = 0.013, **Figure 2A**). Non-CR (*P* = 0.005), positive MRD (*P* = 0.036), and ALC < 500/μl *(P* = 0.014) significantly increased the relapse rate in the univariate analysis. In addition, only non-CR and ALC < 500/μl had statistical significance in the multivariate analysis (*P* = 0.040 and P = 0.025, respectively, **Table 4**).

**Table 4:** Univariate and multivariate analysis of prognostic factors of relapse.

	Univariate Analysis			Multivariate Analysis		
Variable	HR	95% CI	P-value	HR	95% CI	P-value
Sex (male VS female)	0.555	0.236-1.308	0.179			
Age	0.994	0.986-1.002	0.156			
Disease (AML & MDS VS ALL)	1.710	0.640-4.565	0.284			
Disease status (persistent VS remission)	4.178	1.876-9.305	**0.005**	3.045	1.054-8.797	**0.040**
MRD (positive VS negative)	1.954	1.044-3.656	**0.036**	1.369	0.617-3.038	0.439
TBI (positive VS negative)	0.639	0.086-4.765	0.662			
Blood-type match (mismatched vs matched)	1.282	0.544-3.019	0.570			
Donor-recipient sex match (female to male vs others)	0.472	0.110-2.030	0.313			
Graft source (PB+BM vs PB)	1.744	0.638-4.766	0.278			
Graft Dose						
MNC	0.983	0.830-1.164	0.841			
CD34	0.963	0.825-1.123	0.629			
ALC (high VS low)	0.339	0.143-0.806	**0.014**	0.367	0.152-0.883	**0.025**
ALC (continuous)	0.999	0.998-1.000	0.177			

HR, hazard ratio; CI, confidence internal; ALL, acute lymphoblastic leukemia; AML, acute myeloid leukemia; MDS, myelodysplastic syndrome; CR, complete remission; MRD, minimal residual disease; TBI, total body irradiation; PB, peripheral blood; BM, bone marrow; MNC, mononuclear cell ; ALC, Absolute Lymphocyte Counts. P ≤ 0.10 in univariate analysis and P < 0.05 in multivariate analysis. Bold values: P ≤ 0.10 in univariate analysis and P < 0.05 in multivariate analysis.

13 patients died without a history of relapse. Among them, 9 died from infections, 1 from chronic GVHD, 1 from chemotherapy-related toxicity, 1 from renal failure, and 1 had an unknown cause of death. In Gray’s test, the 3-year NRM rates were similar across the two groups (*P* = 0.334, **Figure 2B**).

4 patients died of infection in the low ALC group, and 5 patients in the high ALC group. Infection-related mortality rates were comparable between the two groups (*P* = 0.417). In the low ALC group, six pathogens were detected, including CMV (n = 2), human parvovirus B19 (n = 1), Pseudomonas aeruginosa (n = 2), influenza B (n = 1), and Mycobacterium kansasii (n = 1). In the high ALC group, six pathogens were detected, including *Staphylococcus aureus* (n = 1), human parvovirus B19 (n = 1), Aspergillus fumigatus (n = 1), Pseudomonas aeruginosa (n = 2), Stenotrophomonas maltophilia (n = 1), and COVID-19 (n = 1).

### GVHD

**Figure 3:**
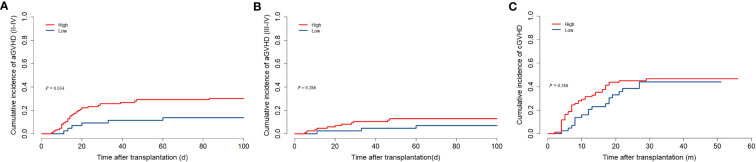
The association between preconditioning absolute lymphocyte counts and GVHD. **(A)** Cumulative incidence of II-IV aGVHD. **(B)** Cumulative incidence of III-IV aGVHD. **(C)** Cumulative incidence of cGVHD.

32 children developed II-IV aGVHD which median onset time was 15 (5–83) days after transplantation. In the low ALC group, II-IV aGVHD was less common than in the high ALC group (30.2% vs. 13.6%, *P* = 0.034, **Figure 3A**). However, the cumulative incidence of III-IV aGVHD was comparable between the two groups (12.8% versus 6.8%, *P* = 0.286, **Figure 3B**). In our study, 57 patients developed cGVHD. Gray’s test showed that the pre-conditioning ALC was not related to cGVHD (*P* = 0.386, **Figure 3C**).

### CMV and EBV reactivation

**Figure 4:**
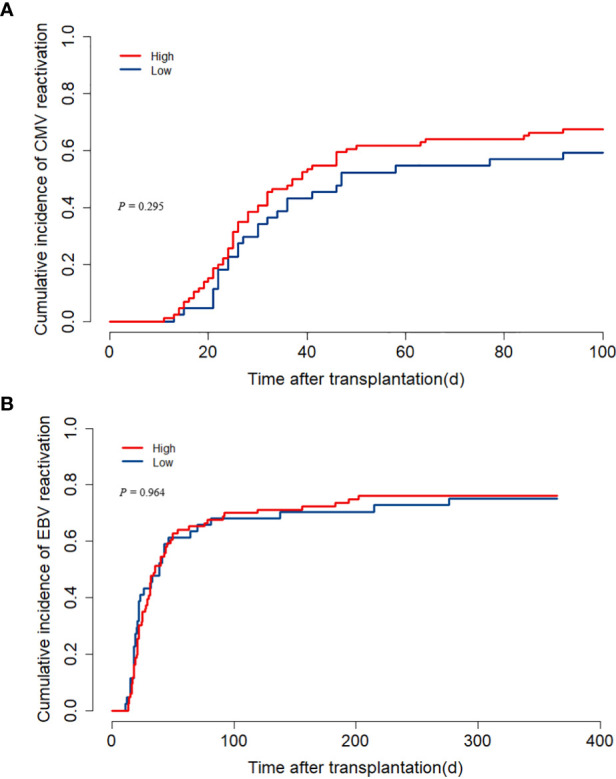
The impact of pre-treatment absolute lymphocyte count (ALC) on CMV and EBV reactivation. **(A)** cumulative incidence of CMV reactivation; **(B)** cumulative incidence of EBV reactivation.

CMV reactivation occurred in 84 children within 100 days after transplantation, and the median onset time was 26.5 (11–92) days. There was no distinction between the two groups (low ALC group versus high group: 43.2% versus 47.7%, *P* = 0.295, **Figure 4A**). Similarly, the two groups showed no substantial distinction in the cumulative incidence of EBV reactivation within one year (low ALC group versus high ALC group: 75.0% versus 76.2%, *P* = 0.964, **Figure 4B**).

### Immune reconstitution

**Figure 5:**
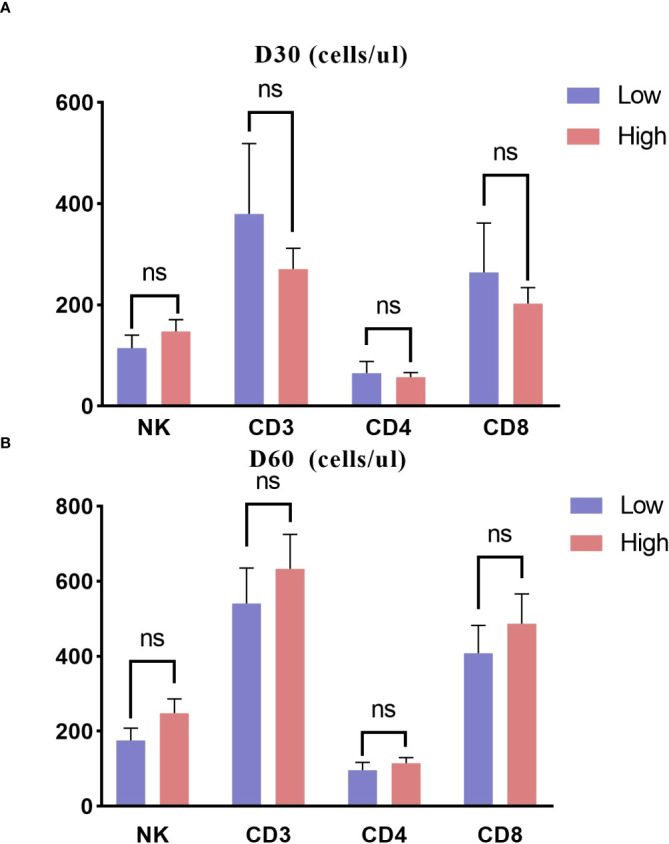
Immune reconstitution about 30 and 60 days posttransplant in two groups. **(A)** D30; **(B)** D60. ns, not significant.

Lymphocyte subset information was available for 78 children on day 30 post-transplant and for 90 children on day 60. The counts of CD3, CD4, CD8, and NK cells on D30 and D60 did not differ substantially among the two groups (**Figures 5A, B**).

### ALC on the day of ATG administration

**Figure 6:**
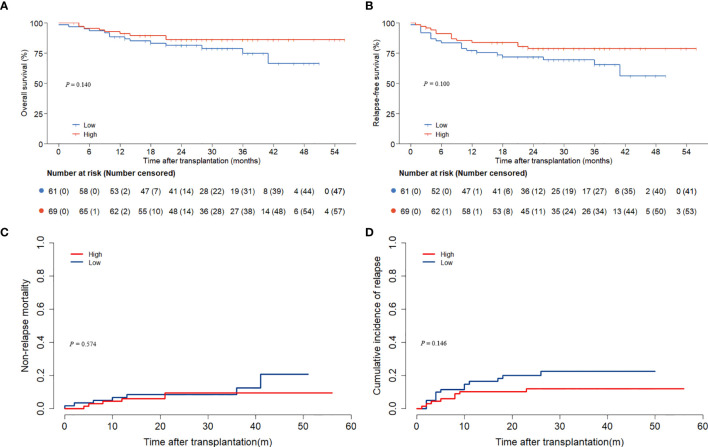
Outcomes according to absolute lymphocyte counts on the day of ATG administration. **(A)** overall survival; **(B)** relapse-free survival; **(C)** non-relapse mortality; **(D)** relapse.

On the day of ATG administration, the median ALC was 30 (0, 930)/μl. We separated the patients into two groups based on the cutoff of 30/μl. The comparison between the two groups revealed no significant differences in OS, RFS, NRM, or relapse (all *P* > 0.05, **Figures 6A–D**). Additionally, a weak positive correlation was found between ALC at the two timepoints. (r = 0.233; *P* = 0.008).

## Discussion

Incorporating ATG in the conditioning regimen decreases the likelihood of GVHD following allo-HSCT. However, excessive ATG administration may result in poorer survival and higher relapses rates (8). ATG clearance is influenced by ALC. Previous studies have shown that higher pre- conditioning ALC can predict better clinical outcomes in HLA-matched HSCT (10, 12, 13). However, the relationship remains unclear in pediatric haplo-HSCT.

ALC < 500 cells/μL is a valuable cutoff, as it is commonly defined as severe lymphopenia and classified as grade ≥3 lymphocytopenia (14). Previous studies have also confirmed the prognostic value of this threshold (10, 12). Furthermore, when we explored higher cutoffs (750 and 1000 cells/μL), the association between ALC and clinical outcomes was weakened, further supporting the significance of the 500 cells/μL threshold.

Our study found that pediatric patients with ALC < 500/μl had lower 3-year OS and RFS, which was consistent with previous research findings (10, 12, 13). In the context of HLA-matched unrelated HSCT, Kennedy et al. showed that in patients with lower ALC, higher ATG doses correlated with higher mortality, but this relationship was reversed in patients with higher ALC (15). Moreover, in a study of 90 haplo-HSCT patients given low-dose ATG as GVHD prophylaxis, those with lower ALC (ALC < 500/μl) exhibited elevated ATG levels in the blood and poorer 1-year OS and RFS (16). High-dose ATG delays post-transplant immune reconstitution (17). However, this does not fully explain the results of our study, as there were no significant differences in immune reconstitution between the two groups after transplantation. We infer that pre-conditioning ALC itself is associated with transplant outcomes. ALC reflects the composition of all lymphocyte lineages, including T cells, B cells, and NK cells, representing the host’s immune robustness. T cell subsets and NK cells can exert anti-leukemic effects (18, 19). In leukemia patients undergoing chemotherapy, the autologous activity of NK cells is a key factor in maintaining sustained remission (18). Ohnishi et al. analyzed the lymphocyte subsets in 30 patients with complete remission of AML, and the results showed that activated T cells and NK cells are crucial for immune surveillance after chemotherapy (20). Therefore, further research is needed to determine whether the ATG dose can be adjusted based on pre-conditioning ALC.

The cumulative incidence of relapses was higher in the low ALC group. The cumulative relapse rate was higher in the low ALC group. A similar result was found in the study by Zhou et al., which showed that patients with pre-conditioning ALC < 500/μl had a higher risk of relapse compared to those with ALC ≥500/μl (33.33% vs. 11.59%, P = 0.041) (16). The association between ALC and NRM remains unclear. In a study involving 84 patients receiving matched related donor HSCT(MRD-HSCT) with a reduced intensity conditioning (RIC), those with ALC < 500/μl had significantly higher NRM rates (28.6% versus 8.6%; *P* = 0.031) (10). However, this phenomenon was not confirmed in the matched unrelated donor HSCT (12).

Infection was the main cause of NRM. Excessive use of ATG led to severe lymphocyte depletion, which was associated with higher infection rates. CMV and EBV reactivation are the most common viral infections in allo-HSCT patients and do harm to prognosis (21, 22). Compared to the standard regime (10mg/kg), reduced ATG dosing has been shown to lower the incidence of CMV and EBV reactivation (23). However, consistent with previous studies, Pre-conditioning ALC had no impact on CMV or EBV reactivation in our study (12, 16). Another common cause of NRM is GVHD. We found that the incidence of II-IV aGVHD was lower in the ALC < 500/μl group, whereas the rates of cGVHD were similar in both groups. Additionally, when focusing on III-IV aGVHD, no notable difference was detected. The higher incidence of aGVHD in haplo-HSCT might account for this observation.

Interestingly, a trend toward better immune reconstitution was observed in the lower ALC group on day 30, despite no significant differences. Post-transplant immune reconstitution is influenced by various factors such as GVHD and viral infections (24). In our study, the high ALC group experienced more aGVHD, and certain immunosuppressive agents used to treat aGVHD, such as steroids, may suppress immune reconstitution (25). CMV viremia is also associated with enhanced T cell reconstitution. Although the reactivation rates of CMV and EBV were similar between the two groups in our study, another study showed that the viral copy numbers were higher in the low ALC group compared to the high ALC group (16). Leserer et al. demonstrated that the level of CD3 T cell reconstitution appears to be proportional to the magnitude of CMV viremia after HSCT (26). However, further investigation is needed to clarify this finding and its underlying mechanisms.

Most current research focuses on the relationship between pre-conditioning ALC or the ALC on the day of ATG administration and clinical outcomes, showing that patients with higher ALC tend to have better prognoses. In this study, we found that only pre-conditioning ALC was significantly associated with improved clinical outcomes, while ALC on the day of ATG administration showed no correlation. This discrepancy may be related to the lower median ALC on the day of ATG administration in our study compared to previous reports, such as Modi et al. (200/μl, range = 100–6000/μl) (13) and Shiratori et al. (840/μl, range = 0–880/μl) (27). However, further studies are needed to clarify this difference.

Some limitations should be addressed in our study. First, as a single-center retrospective study with a limited number of patients, the accuracy of the results may be constrained. Second, our institution does not perform dynamic monitoring of ATG concentration after transplantation. Third, lymphocyte subset data beyond 3 months post-transplant is limited, preventing further comparison of immune reconstitution differences.

In conclusion, our study suggested that pre-conditioning ALC was a significant prognostic factor for OS, RFS and relapses in pediatric patients undergoing MAC-HSCT. However, whether the ATG dose should be adjusted based on pre-conditioning ALC remains to be explored in future studies.

## Data Availability

The raw data supporting the conclusions of this article will be made available by the authors, without undue reservation.
