# Supplementary material for: Lower pre-conditioning absolute lymphocyte counts are associated with worse outcomes in haploidentical stem cell transplantation with myeloablative regimen in children
Source: Front Immunol. 2025 Mar 25;16:1552263. doi: 10.3389/fimmu.2025.1552263 (PMC11975963; doi:10.3389/fimmu.2025.1552263)

Supplemental Materials

Figure S1

Overall survival based on different cutoff values for pre-conditioning absolute lymphocyte counts. (A: 750/μL; B: 1000/μL)

A.


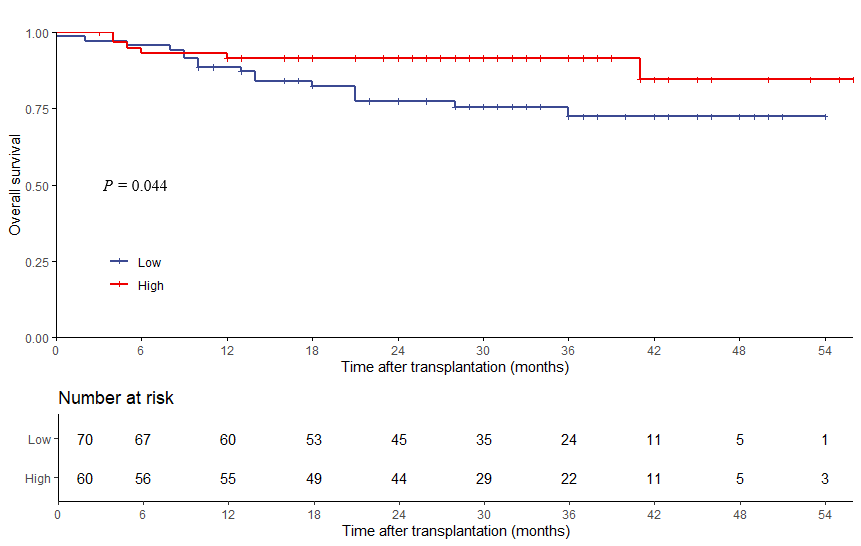


B.


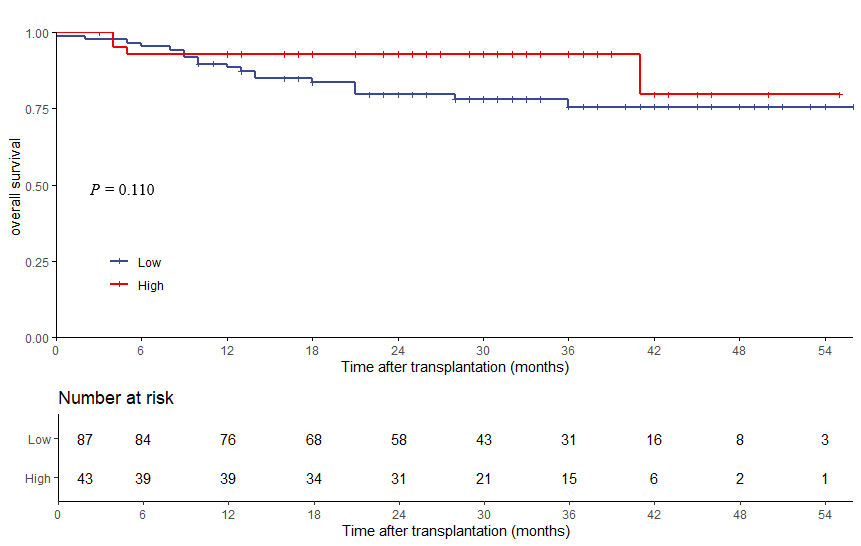


Figure S2

Relapse-free survival based on different cutoff values for pre-conditioning absolute lymphocyte counts. (A: 750/μL; B: 1000/μL)

A.


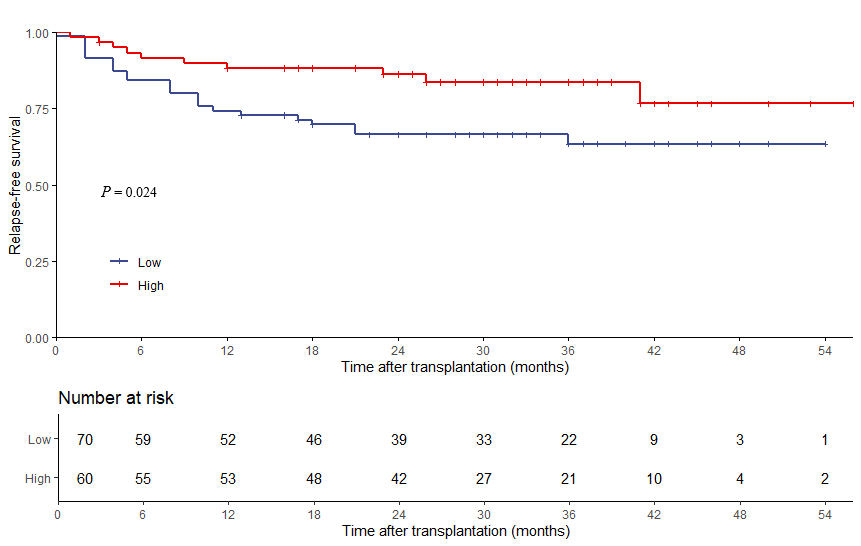


B.


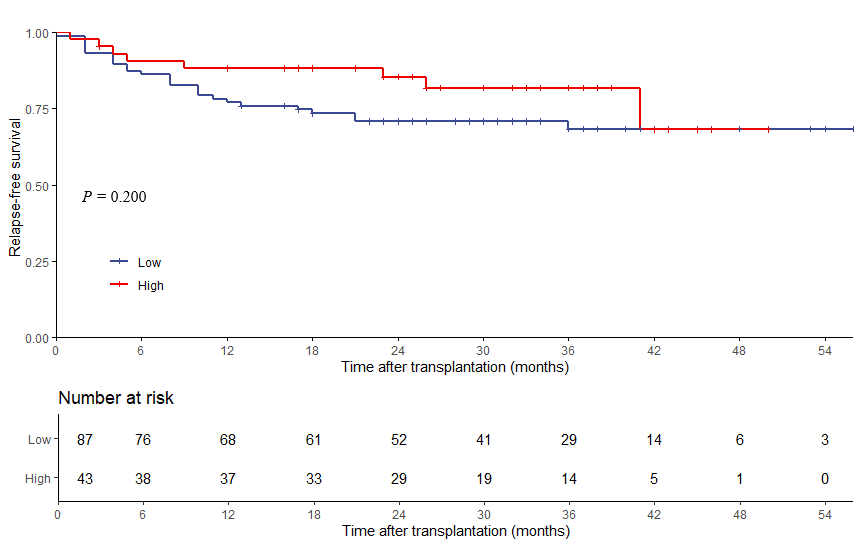


Figure S3

The cumulative incidence of relapse based on different cutoff values for pre-conditioning absolute lymphocyte counts. (A: 750/μL; B: 1000/μL)

A.


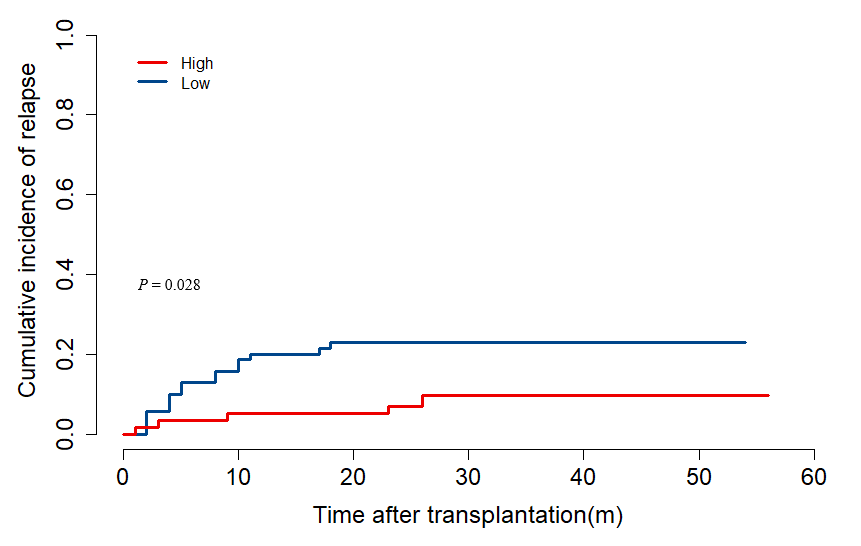


B.


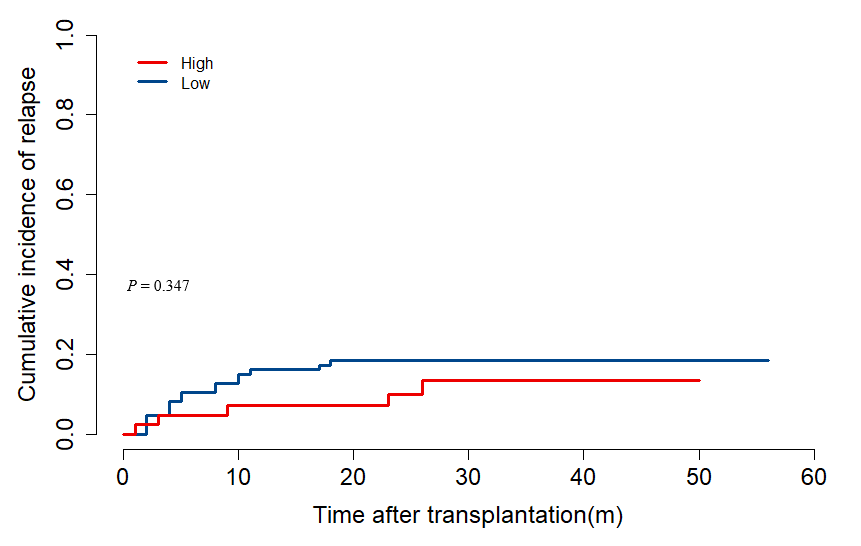


Figure S4

The cumulative incidence of II-IV aGVHD based on different cutoff values for pre-conditioning absolute lymphocyte counts. (A: 750/μL; B: 1000/μL)

A.


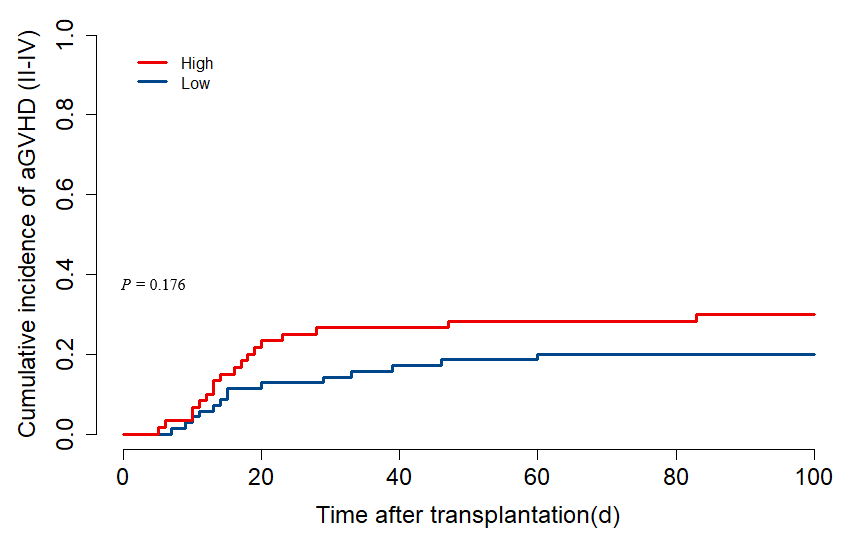


B.


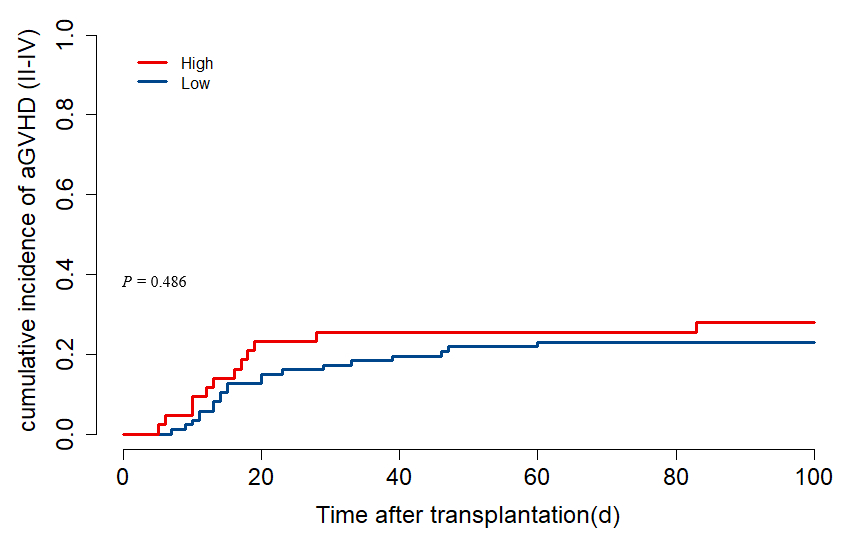


Figure S5

The cumulative incidence of III-IV aGVHD based on different cutoff values for pre-conditioning absolute lymphocyte counts. (A: 750/μL; B: 1000/μL)

A.


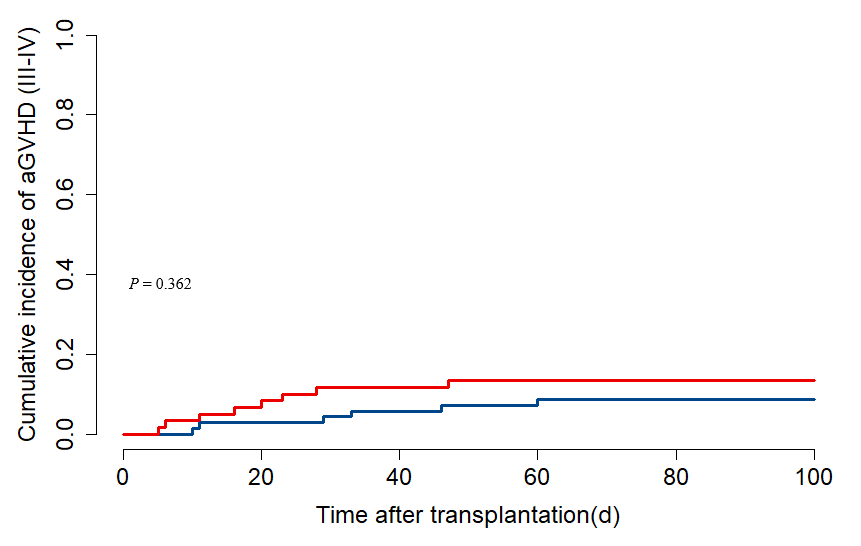


B.


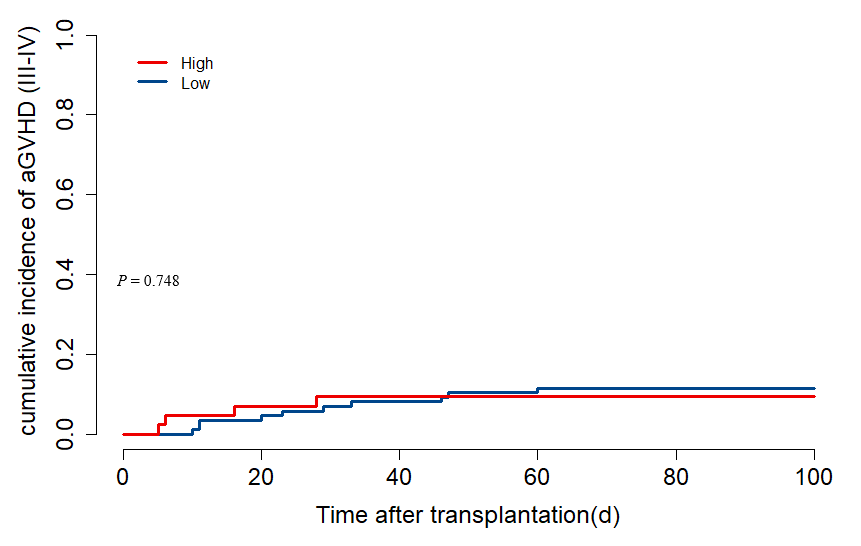


Figure S5

The cumulative incidence of non-relapse mortality based on different cutoff values for pre-conditioning absolute lymphocyte counts. (A: 750/μL; B: 1000/μL)

A.
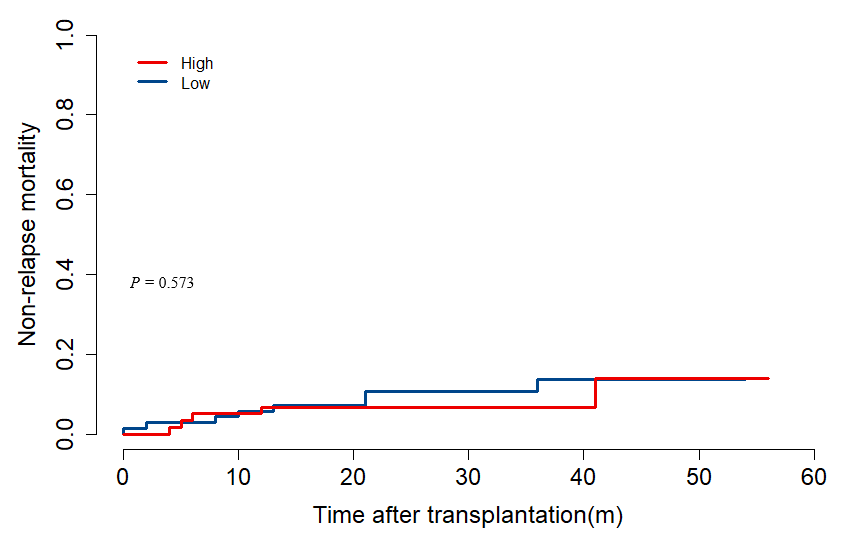


B.


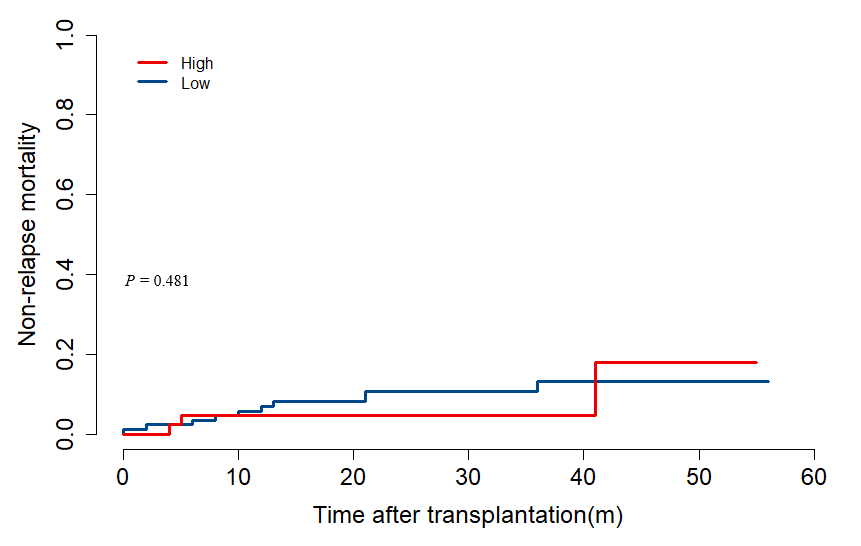

Supplement: Supplementary file 1 [file DataSheet1.docx]
